# Dysbiosis of gut microbiota inhibits NMNAT2 to promote neurobehavioral deficits and oxidative stress response in the 6-OHDA-lesioned rat model of Parkinson’s disease

**DOI:** 10.1186/s12974-023-02782-1

**Published:** 2023-05-19

**Authors:** Jianjun Yu, Jianhong Meng, Zhengwei Qin, Yuan Yu, Yingxin Liang, Yanjun Wang, Dongmei Min

**Affiliations:** 1 Orthopedics of Chinese Medicine, The Fourth Affiliated Hospital of Guangxi Medical University, Liuzhou, 545000 People’s Republic of China; 2 Department of Acupuncture, Heilongjiang Academy of Chinese Medical Sciences, Harbin, 150036 People’s Republic of China; 3 Department of Massage, Heilongjiang Academy of Chinese Medical Sciences, Harbin, 150036 People’s Republic of China; 4 Department of Rehabilitation Medicine, The Fourth Affiliated Hospital of Guangxi Medical University, No. 156, Heping Road, Liunan District, Liuzhou, 545000 Guangxi Zhuang Autonomous Region People’s Republic of China

**Keywords:** Parkinson’s disease, Dysbiosis of gut microbiota, Fecal microbiota transplantation, NMNAT2, Neurobehavioral symptoms, Oxidative stress response

## Abstract

**Background:**

New data are accumulating on gut microbial dysbiosis in Parkinson’s disease (PD), while the specific mechanism remains uncharacterized. This study aims to investigate the potential role and pathophysiological mechanism of dysbiosis of gut microbiota in 6-hydroxydopamine (6-OHDA)-induced PD rat models.

**Methods:**

The shotgun metagenome sequencing data of fecal samples from PD patients and healthy individuals were obtained from the Sequence Read Archive (SRA) database. The diversity, abundance, and functional composition of gut microbiota were further analyzed in these data. After the exploration of the functional pathway-related genes, KEGG and GEO databases were used to obtain PD-related microarray datasets for differential expression analysis. Finally, in vivo experiments were performed to confirm the roles of fecal microbiota transplantation (FMT) and upregulated NMNAT2 in neurobehavioral symptoms and oxidative stress response in 6-OHDA-lesioned rats.

**Results:**

Significant differences were found in the diversity, abundance, and functional composition of gut microbiota between PD patients and healthy individuals. Dysbiosis of gut microbiota could regulate NAD^+^ anabolic pathway to affect the occurrence and development of PD. As a NAD^+^ anabolic pathway-related gene, NMNAT2 was poorly expressed in the brain tissues of PD patients. More importantly, FMT or overexpression of NMNAT2 alleviated neurobehavioral deficits and reduced oxidative stress in 6-OHDA-lesioned rats.

**Conclusions:**

Taken together, we demonstrated that dysbiosis of gut microbiota suppressed NMNAT2 expression, thus exacerbating neurobehavioral deficits and oxidative stress response in 6-OHDA-lesioned rats, which could be rescued by FMT or NMNAT2 restoration.

## Background

Parkinson’s disease (PD) is a common neurodegenerative disorder [1–4]. Owing to dopaminergic neuron degeneration in the substantia nigra (SN), the main feature of PD is the deterioration of motor activities [5, 6]. PD patients often suffer from motor symptoms (resting tremor, bradykinesia, muscle tone rigidity, and postural instability) and also non-motor symptoms (sensory abnormalities, sleep disorders, and autonomic dysfunctions) [7, 8]. Interestingly, oxidative stress is among the main factors behind neurodegeneration in PD [9–12].

Of note, emerging evidence suggests that gut microbiota is associated with PD [13–15]. Gut microbiota, known as “forgotten organ”, is home to 100 trillion bacteria, 10-fold the number of cells in the human body [16, 17]. The host relies on its gut microbiota for many important functions, which is crucial for the regulation of health [18, 19]. Fecal microbiota transplantation (FMT) can directly alter recipients’ gut microbiota for normalizing the composition and gaining therapeutic benefits [20, 21]. In recent years, dysbiosis of gut microbiota in PD has attracted great attention. For instance, a distinctive microbiota composition was found in PD patients, accompanied by alteration in pathways regulating the pro-inflammatory environment in the gut and a decline in amino acid biosynthesis [22]. In addition, dysbiosis of gut microbiota contributes to increased oxidative stress in PD [23, 24]. However, the possible mechanism between gut microbial dysbiosis and neurobehavioral symptoms and oxidative stress response of PD remains elusive, with no therapies recognized to delay the progression of PD.

Furthermore, bioinformatics analysis in this study revealed that nicotinamide mononucleotide adenylyl-transferase 2 (NMNAT2) was differentially expressed in PD patients. NMNAT2, one of the isoforms of NMNAT, is a key enzyme that catalyzes the synthesis of nicotinamide adenine dinucleotide (NAD^+^) from NMN [25]. NMNAT2 is a newly identified factor for maintaining neuron function, and NMNAT2 expression is positively related to cognitive function and negatively associated with pathological features of neurodegenerative diseases [26]. It should also be noted that NMNAT2 is involved in mitochondrial dysfunction to affect the progression of PD [27]. Herein, from the aforementioned findings, we might hypothesize that dysbiosis of gut microbiota could affect the neurobehavioral symptoms and oxidative stress in 6-hydroxydopamine (6-OHDA)-lesioned rats by mediating NMNAT2. Therefore, we undertook bioinformatics analysis and in vivo experiments in the PD model to illuminate a more efficacious personalized treatment strategy for PD by regulating dysbiosis of gut microbiota-mediated NMNAT2.

## Methods

### Data retrieval

The NCBI Sequence Read Archive (SRA) database was used to acquire the published PD-related shotgun metagenome sequencing data ERP019674. It included fecal samples from male patients with early-stage PD who received no drugs (*n* = 31) and age-matched healthy individuals (*n* = 28).

### Analysis of microbial abundance

The samples were evaluated using multiQC and kneaddata, in which multiQC was used for sequence quality control and kneaddata for removing host and contamination sequences. The GraPhlAn was employed to plot the microbial species tree and annotate the difference to obtain the relative abundance of the microbial classification. To assess the diversity and complexity of species in the samples, Alpha diversity was analyzed by the invsimpson index and beta diversity based on the principal coordinate analysis (PCoA). Wilcoxon rank-sum and Welch *t* tests were applied for the comparison of bacterial abundance and diversity. The linear divergence analysis (LDA) effect size was performed to analyze and draw a differential abundance histogram with an LDA score of 2.0 as the threshold. LDA scores indicate the influence degree of significantly differentially abundant species between different groups, and the higher scores indicate more significant differences in characteristics between the two groups.

### Microbial functional composition

The HUMAnN2 was used to obtain the tables of pathway abundance, including functional pathways and species composition, followed by the acquisition of stratified (relative abundance of unclassified species) and unstratified (relative abundance of classified species) results. The unstratified results were visualized by the STAMP software (v2.1.3). The Welch *t* test was employed to compare the functional composition.

### Differentially expressed gene (DEG) analysis

The pathway-related products and related genes were obtained through the Kyoto Encyclopedia of Genes and Genomes (KEGG) database. PD-related microarray datasets GSE7621, GSE20168, and GSE20291 were acquired by the Gene Expression Omnibus (GEO) database. The GSE7621 dataset consisted of SN tissue samples from 16 PD patients and 9 healthy individuals with GPL570 as the microarray sequencing platform file. The GSE20168 dataset included prefrontal cortex area 9 samples from 14 PD patients and 15 healthy individuals with GPL96 as microarray sequencing platform file. The GSE20291 dataset was composed of putamen tissue samples from 15 PD patients and 20 healthy individuals with GPL96 as a microarray sequencing platform file. Gene expression profiles were normalized by the R “limma” package, using |logFC|> 1 and *p* value < 0.05 as the screening criteria for DEGs.

### Experimental animals

This study protocol was approved by the Experimental Animal Ethics Committee of The Fourth Affiliated Hospital of Guangxi Medical University. Adult Wistar male rats weighing between 230 and 260 g from Hunan SJA Laboratory Animal Co., Ltd. (Changsha, China) were reared in a specific pathogen-free (SPF) laboratory (60–65% humidity, 22–25 °C), free access to sterilized water and food. The experiment was conducted after acclimatization for 1 week.

The rats were sham operated (injected with normal saline) or induced by 6-OHDA. The 6-OHDA-lesioned rats were untreated or subjected to further treatment with lentiviral vectors containing NMNAT2 (oe-NMNAT2), FMT, or lentiviral vectors containing short hairpin RNA (sh)-NMNAT2 + FMT. Eight rats were included in each group. Lentiviruses were purchased from Genechem Co., Ltd. (Shanghai, China). The gene overexpression vector Lenti-CMV-EGFP-P2A-3FLAG-Rac1, gene silencing vector LV-CX36-shRNA-EGFP, and 6-OHDA were purchased from Sigma.

The 6-OHDA was used to induce dopamine neuronal degeneration for the construction of PD rat models [28]. Next, rats were anesthetized via intraperitoneal injection of the mixture of 40 mg/kg pentobarbital sodium (Sigma), and then fixed on a stereotaxic device. Based on the brain atlas of Paxinos and Watson, at the coordinates relative to the anterior fontanelle and dura mater, a Hamilton syringe (Hamilton, Reno, NV) was used to inject lentivirus (9 × 10^8^ TU/mL) carrying overexpression or NC vectors into SN (AP-5.3, ML-2.2, DV-7.2) at a rate of 0.2 µL/min and inject 6-OHDA (10 µg) into striatum (AP 0, ML-2.6, DV-5 and AP-1.2, ML-3.9, DV-5) at a rate of 0.4 µL/min. The needle was left in place for 5 min, before it was slowly pulled back.

FMT treatment: the fresh fecal pellet was harvested from sham-operated rats (donor rats), and 6-OHDA-treated rats (receptor rats) were given intragastric gavage every day for 2 weeks. In brief, fecal pellets from donor rats in the cage were collected, diluted in sterile phosphate-buffered saline (PBS; 1 fecal pellet/3 mL), immersed for 20 min, and shaken. The fecal pellets were centrifuged at 500 g for 30 s to obtain the soluble fraction. For receptor rats, one week after injection with 6-OHDA, rats were administered 2 mL of suspension by gavage, followed by transplantation once every day for two weeks.

At the 2nd and 3rd week following injection with 6-OHDA, rats were subjected to behavioral tests. The rats were euthanized for biochemical studies.

### Behavioral observations

Apomorphine-induced rotation test: Drug-induced rotational behavior has conventionally been used to determine the extent of unilateral lesions. Each rat was injected with 0.5 mg/kg apomorphine (QCCQA16150010MG, Sinopharm Chemical Reagent Co., Ltd. Shanghai, China) intraperitoneally. After 5 min, the rotation behaviors of rats were observed and recorded. Rats rotated to the opposite side of the lesion with the contralateral hindlimb as the fulcrum. The tail and head were connected, and the body was bent and rotated in a circle. The number of contralateral rotations within 30 min was calculated for each group.

Open field test: Rats were placed in an open field test box with length, width and height of 100 × 100 × 40 cm for 5 min. The moving distance and speed of rats were analyzed. The video recording and analysis of the behavioral test were performed using the Ethovision XT8.5 software.

Rotation test: Rotation test was used to assess motor coordination of rats. Rats were placed on a rotary rod with a diameter of 7 cm. The rotation speed was increased from 5 r/min to 35 r/min within 5 min. The residence time of the rat on the rotating shaft was recorded as the latency (the time from the beginning of rotation to the fall of rats). Each rat was tested 3 times to obtain the average value, and each experiment lasted 5 min with an interval of 30 min.

### Tissue collection

Rats were deeply anesthetized with 120 mg/kg pentobarbital sodium (Sigma) via intraperitoneal injection. The left ventricular perfusion was performed with 0.9% saline (Sinopharm Chemical Reagents, Shanghai, China) and 4% paraformaldehyde (Wuhan Service Biotechnology Co., Ltd., Wuhan, China). Brains were harvested, fixed, frozen in 30% sucrose solution and sliced into coronal section (30 µm thick) using a cryoslicer (Leica, Wetzlar, Germany). For biochemical studies, the midbrain and striatum were separated on ice and rapidly frozen in liquid nitrogen.

### Immunohistochemistry (IHC)

The brain tissue sections were treated with endogenous peroxidase blocker (PV6001, Zhongshan Golden Bridge, Beijing, China) to eliminate the endogenous peroxidase. Next, the sections were blocked in PBS containing 10% normal goat serum and 0.3% Triton-X 100, and probed with the rabbit antibody against Anti-Tyrosine Hydroxylase (TH; ab6211, 1: 500, Abcam Inc., Cambridge, UK) or NMNAT2 (PA5-115662, 1: 100, Invitrogen, Carlsbad, CA) and then with goat anti-rabbit IgG (HRP-conjugated, Zhongshan Golden Bridge). Subsequently, the sections were developed with DAB (Zhongshan Golden Bridge) and observed using a bright-field microscope (DM2500, Leica). The total number of TH-positive cells in the substantia nigra was counted.

### Enzyme-linked immunosorbent assay (ELISA)

NMNAT2 expression was measured using the rat ELISA detection kit (abx535445, Abbexa, Cambridge, UK). The samples were added with biotinylated anti-rat NMNAT2, and the unbound biotinylated antibodies were washed. Subsequently, the samples were added with HRP-conjugates and TMB substrate. With the addition of TMB substrate, the optical density (OD)_450_ value was measured.

### Determination of malondialdehyde (MDA), glutathione (GSH), total superoxide dismutase (SOD), and glutathione peroxidase (GSH-Px)

The lipid peroxidation, GSH content, SOD activity, and GSH-Px activity in tissues were assessed using the MDA detection kit (A003-1), the GSH detection kit (A006-2), the total SOD assay kit (A001-1), and the GSH-Px assay kit (A005-1). These kits were from Jiancheng Bioengineering Institute (Nanjing, China) [29–31].

### Statistical analysis

Data were analyzed by the R software v3.6.0 (R Foundation for Statistical Computing, Vienna, Austria) and SPSS 21.0. (IBM Corp. Armonk, NY). All quantitative data are presented as mean ± standard deviation. The data between two groups were analyzed by unpaired *t*-test, and multi-group data comparison was performed by one-way analysis of variance (ANOVA) with Tukey’s post hoc test. *p* < 0.05 reflects statistical significance.

## Results

### There are differences in gut microbiota diversity between PD patients and healthy individuals

**Fig. 1:**
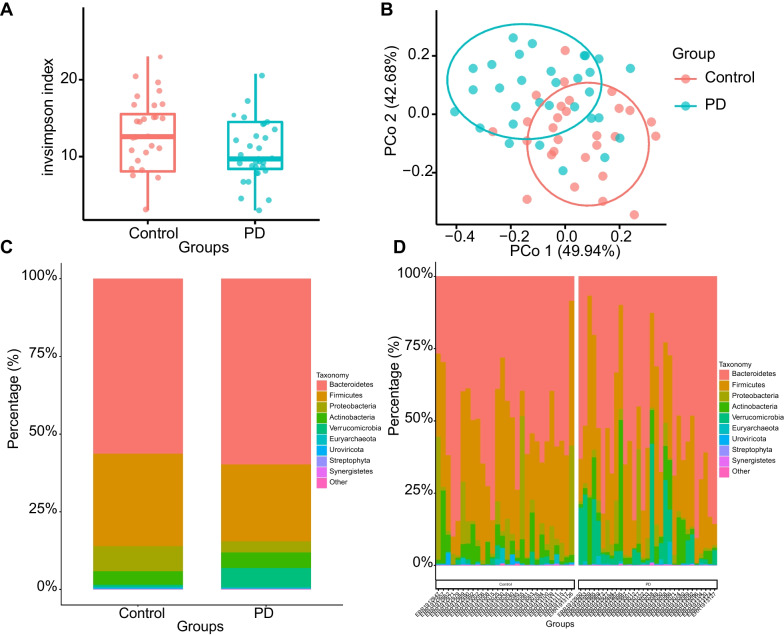
Comparison of species diversity of gut microbiota between PD patients and healthy individuals. **A** Alpha diversity analysis of gut microbiota in samples from PD patients (*n* = 31) and healthy individuals (*n* = 28). **B** Beta diversity analysis of gut microbiota in samples from PD patients (*n* = 31) and healthy individuals (*n* = 28). **C** Quantitative analysis of relative abundance at the phylum level with the group as the horizontal axis. Different colors represent the gut microbiota of different phyla. **D** Quantitative analysis of the relative abundance of the main samples in PD patients (*n* = 31) and healthy individuals (*n* = 28). Different colors represent the gut microbiota of different phyla. *p < 0.05

We first assessed gut microbiota diversity differences between PD patients and healthy individuals. The sequences were aligned for the estimation of alpha and beta diversity. Alpha diversity can be used to calculate species composition within samples, including two-dimensional information on number and abundance, while beta diversity can be used to study species composition among communities [32]. The alpha diversity analysis showed that the invsimpson index varied significantly between the two groups (Fig. 1A).

The beta diversity distance matrix was calculated using three common similarity/distance indices (bray_curtis, jaccard, and manhatten). The PCoA analysis was conducted based on the bray_curtis distance, which demonstrated a significant separation between the two groups (Fig. 1B). In addition, species composition analysis suggested that the species composition of gut microbiota between PD patients and healthy individuals varied significantly at the phylum level (Fig. 1C, D). *Firmicutes* and *Proteobacteria* were more abundant in healthy individuals, and *Verrucomicrobia* was more abundant in PD patients.

These findings indicated significant differences in gut microbiota diversity between PD patients and healthy individuals.

### There are differences in gut microbiota composition between PD patients and healthy individuals

**Fig. 2:**
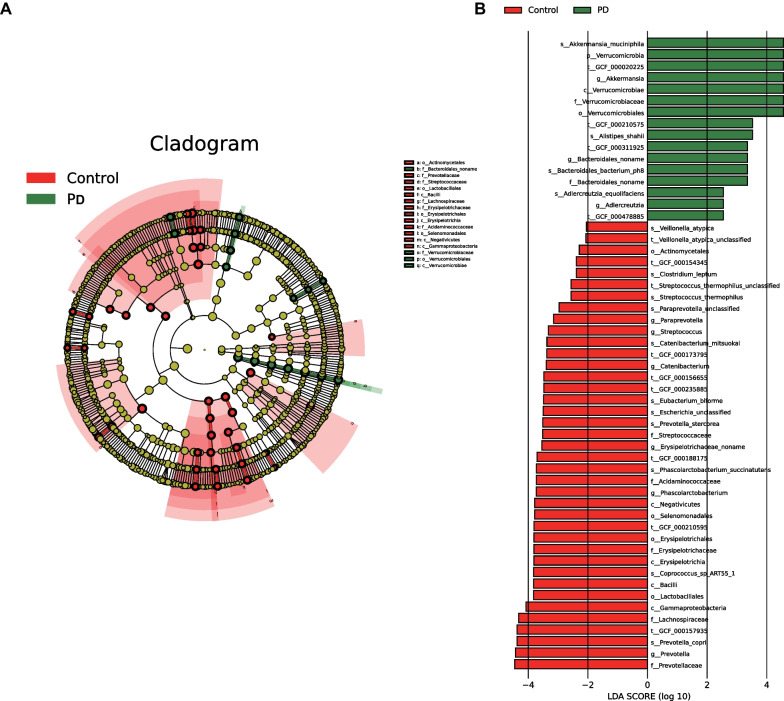
Difference in gut microbiota composition between PD patients and healthy individuals. **A** Branch plot showing the classification of species abundance of gut microbiota in PD patients (*n* = 31) and healthy individuals (*n* = 28). The circle radiating from inside to outside represents the classification level from phyla to genus, and the diameter represents the size of relative abundance. The yellow nodes indicate species without significant differences, the red nodes indicate species with higher abundance in healthy individuals, and the blue nodes indicate species with higher abundance in PD patients. **B** Histogram showing LDA distribution of species abundance of gut microbiota in PD patients (*n* = 31) and healthy individuals (*n* = 28)

Next, the specific composition difference of the gut microbiota was further investigated between the two groups. A LEfSe analysis was performed to visualize the results. It was found that the relative abundance of *Akkermansia muciniphila*, *Akkermansia*, *Verrucomicrobia*, *Verrucomicrobiae*, *Verrucomicrobiaceae*, *Verrucomicrobiales*, *Alistipes shahii*, *Bacteroidales noname*, *Bacteroidales bacterium*, *Adlercreutzia equolifaciens*, and *Adlercreutzia* in the fecal samples from PD patients was higher versus that in the healthy individuals (LDA score (log10) > 2); whereas, *Prevotellaceae*, *Prevotella*, *Prevotella copri*, *Lachnospiraceae*, *Gammaproteobacteria*, *Lactobacillales*, *Bacilli*, *Coprococcus*, *Erysipelotrichia*, *Erysipelotrichaceae*, *Erysipelotrichales*, *Selenomonadales*, *Negativicutes*, *Phascolarctobacterium*, *Acidaminococcaceae*, *Phascolarctobacterium succinatutens*, *Erysipelotrichaceae noname*, *Streptococcaceae*, *Prevotella stercorea*, *Eubacterium biforme*, *Catenibacterium mitsuokai*, *Streptococcus*, *Paraprevotella*, *Streptococcus thermophilus*, *Clostridium leptum*, *Actinomycetales*, and *Veillonella atypica* were more abundant in fecal samples from healthy individuals (LDA score (log10) > 2) (Fig. 2A, B). Therefore, gut microbiota composition significantly differed between PD patients and healthy individuals. The above differentially abundant microbiota could distinguish the microbiota of healthy individuals and PD patients.

### There are differences in the functional composition of gut microbiota between PD patients and healthy individuals

**Fig. 3:**
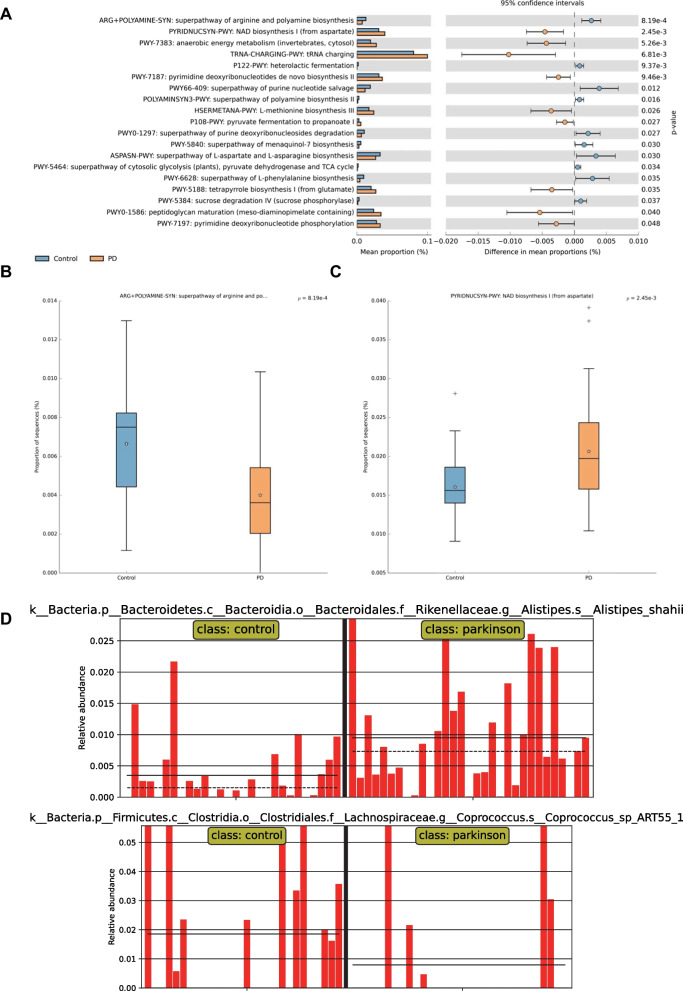
Functional analysis of the gut microbiota in PD patients and healthy individuals. **A** Functional analysis of gut microbiota in PD patients (*n* = 31) and healthy individuals (*n* = 28). **B** Difference in superpathway of arginine and polyamine biosynthesis in the samples from PD patients (*n* = 31) and healthy individuals (*n* = 28). **C** Difference in NAD biosynthesis I (from aspartate) in the samples from PD patients (*n* = 31) and healthy individuals (*n* = 28). **D** Difference in the abundance of *Alistipes shahii* and *Coprococcus* in the samples from PD patients (*n* = 31) and healthy individuals (*n* = 28). * *p* < 0.05

The difference in functional composition of gut microbiota between PD patients and healthy individuals was further explored, which was visualized by the STAMP software. As shown in Fig. 3A–C, gut microbiota was mainly enriched in the superpathway of arginine and polyamine biosynthesis and NAD biosynthesis I (NAD^+^ anabolic pathway) functional pathways. Previous studies have revealed that the polyamine pathway and NAD and its associated metabolites are essential for the occurrence and progression of PD [33, 34]. Furthermore, the difference in bacteria associated with NAD^+^ anabolic pathways was also found. *Alistipes shahii* had a higher abundance in PD patients, and *Coprococcus* had a higher abundance in healthy individuals (Fig. 3D). Cumulatively, gut microbiota may participate in the occurrence and development of PD through superpathway of arginine and polyamine biosynthesis and NAD^+^ anabolic pathways.

### Dysbiosis of gut microbiota is involved in the development of PD by inhibiting NMNAT2 expression

Based on the findings described above, we moved to investigate the metabolites and related genes of the superpathway of arginine and polyamine biosynthesis and NAD^+^ anabolic pathways in the KEGG database. It was found that the end product of the polyamine synthesis pathway was spermidine. Previous evidence has reported that spermidine attenuates rotenone-induced dopaminergic neuron loss, oxidative stress, and neuroinflammation in PD rat models, suggesting a promising neuroprotective role [35]. Therefore, we focused on the effects of the NAD^+^ anabolic pathway on PD.

**Fig. 4:**
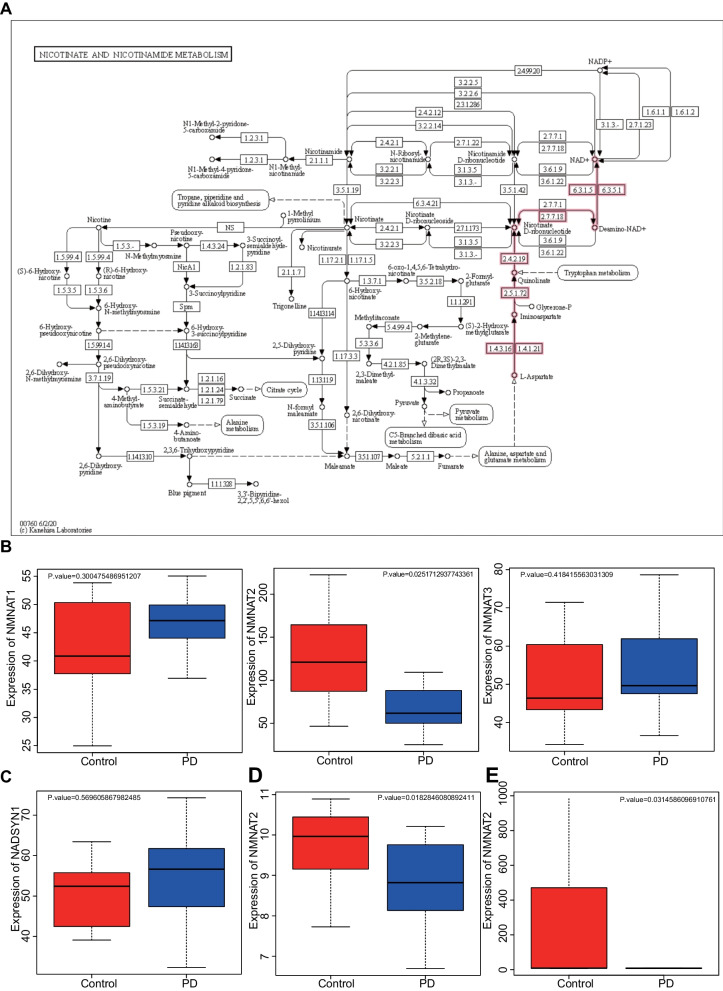
Expression of NADSYN1 and NMNAT in different regions in PD patients. **A** Nicotinic acid and nicotinamide metabolic pathways in KEGG database (map00760, NAD^+^ anabolic pathway in red, M00115). **B** Differential mRNA expression of NMNAT1, NMNAT2, and NMNAT3 in PD patients (*n* = 16) and healthy individuals (*n* = 9) in microarray dataset GSE7621. **C** Differential expression of NADSYN1 mRNA in PD patients (*n* = 16) and healthy individuals (*n* = 9) in microarray dataset GSE7621. **D** Differential expression of NMNAT2 mRNA in PD patients (*n* = 14) and healthy individuals (*n* = 15) in microarray dataset GSE20168. **E** Differential expression of NMNAT2 mRNA of in PD patients (*n* = 15) and healthy individuals (*n* = 20) in microarray dataset GSE20291

Moreover, the key genes in the NAD^+^ anabolic pathway were NADSYN1 and NMNAT, encoding the key catalytic enzymes of the pathway, NAD^+^ synthase and nicotinamide mononucleotide adenosine transferase, respectively (M00115, Fig. 4A). The microarray dataset GSE7621 was used for the analysis of mRNA levels of NADSYN1 and NMNAT in the SN of PD patients, which showed decreased NMNAT2 mRNA level in the SN of PD patients, while the mRNA levels of NADSYN1, NMNAT1, and NMNAT3 were not significantly different (Fig. 4B, C). In addition, NMNAT2 mRNA level was also reduced in the prefrontal cortex area 9 and putamen area of PD patients in microarray datasets GSE20168 and GSE20291 (Fig. 4D, E). The obtained data revealed that dysbiosis of gut microbiota inhibited NMNAT2 expression to affect the occurrence and development of PD.

### FMT alleviates neurobehavioral deficits and oxidative stress response in 6-OHDA-lesioned rats

**Fig. 5:**
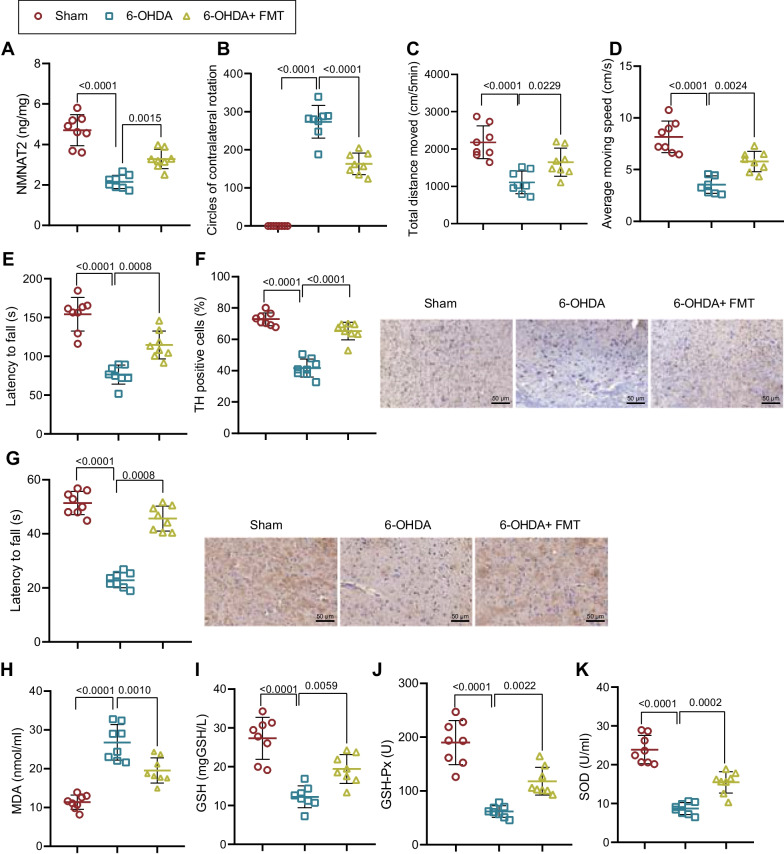
Effects of FMT on neurobehavioral symptoms and oxidative stress response in 6-OHDA-lesioned rats. PD rat models were induced by 6-OHDA, followed by treatment with FMT (*n* = 8). **A** Expression of NMNAT2 in brain tissues of 6-OHDA-lesioned rats detected by ELISA. **B** Number of rotations of 6-OHDA-lesioned rats measured by apomorphine-induced rotation test. **C** Total distance of movement of 6-OHDA-lesioned rats measured by open field test. **D** Average speed of movement of 6-OHDA-lesioned rats measured by open field test. **E** Latency period of 6-OHDA-lesioned rats falling from the rotating rod measured by rotation test. **F** Dopaminergic neurons in the SN of 6-OHDA-lesioned rats detected by IHC. **G** NMNAT2 protein expression in the SN of 6-OHDA-lesioned rats detected by IHC. **H** MDA content in the brain tissues of 6-OHDA-lesioned rats. **I** GSH content in the brain tissues of 6-OHDA-lesioned rats. **J** GSH-Px activity in the brain tissues of 6-OHDA-lesioned rats. **K** SOD activity in the brain tissues of 6-OHDA-lesioned rats. *p < 0.05; **p < 0.01; ***p < 0.001

The mechanistic prediction described above intrigued us to further investigate the effects of microbiota transplantation on PD in rats. The ELISA data suggested that the NMNAT2 expression was diminished in the brain tissues of 6-OHDA-lesioned rats, while further treatment with FMT increased NMNAT2 expression (Fig. 5A). Behavioral observations demonstrated that the number of rotations, moving distance and speed, and rod latency were decreased in 6-OHDA-lesioned rats, while these effects were counteracted by further treatment with FMT (Fig. 5B–E).

Moreover, IHC revealed that TH-positive neurons and NMNAT2 protein expression were decreased in SN of 6-OHDA-lesioned rats, while the effects were counteracted by further treatment with FMT (Fig. 5F, G). Additionally, it was also found that 6-OHDA induction elevated MDA content and reduced GSH content, GSH-Px activity, and SOD activity in brain tissues of 6-OHDA-lesioned rats, while further treatment with FMT exerted opposite effects (Fig. 5H–K). These findings indicated that FMT upregulated NMNAT2 expression, thereby relieving neurobehavioral deficits and oxidative stress in 6-OHDA-lesioned rats.

### Overexpression of NMNAT2 relieves neurobehavioral deficits and oxidative stress response in 6-OHDA-lesioned rats

**Fig. 6:**
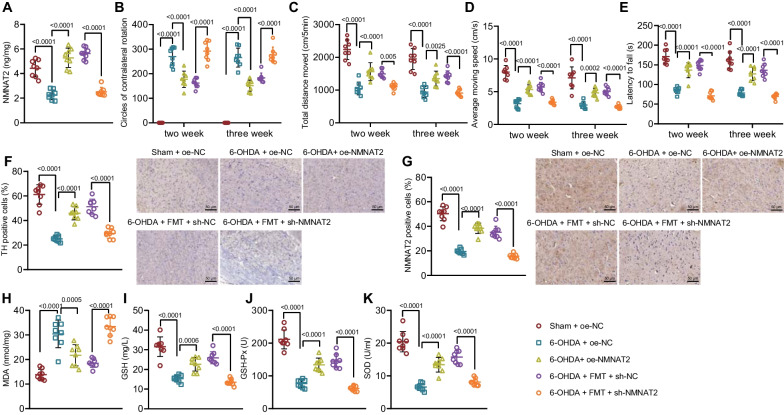
Effects of upregulation of NMNAT2 on neurobehavioral symptoms and oxidative stress response in 6-OHDA-lesioned rats. PD rat models were induced by 6-OHDA, followed by treatment with oe-NMNAT2, FMT or sh-NMNAT2 + FMT (*n* = 8). **A** Expression of NMNAT2 in brain tissues of 6-OHDA-lesioned rats detected by ELISA. **B** Number of rotations of 6-OHDA-lesioned rats measured by apomorphine-induced rotation test. **C** Total distance of movement of 6-OHDA-lesioned rats measured by open field test. **D** Average speed of movement of 6-OHDA-lesioned rats measured by open field test. **E** Latency period of 6-OHDA-lesioned rats falling from the rotating rod measured by rotation test. **F** Dopaminergic neurons in the SN of 6-OHDA-lesioned rats detected by IHC. **G** NMNAT2 protein expression in the SN of 6-OHDA-lesioned rats detected by IHC. **H** MDA content in the brain tissues of 6-OHDA-lesioned rats. **I** GSH content in the brain tissues of 6-OHDA-lesioned rats. **J** GSH-Px activity in the brain tissues of 6-OHDA-lesioned rats. **K** SOD activity in the brain tissues of 6-OHDA-lesioned rats. *p < 0.05; **p < 0.01; ***p < 0.001

Finally, we further verified the role of NMNAT2 in 6-OHDA-lesioned rat models in response to oe-NMNAT2. ELISA data showed that NMNAT2 expression was reduced in the brain tissues of 6-OHDA-lesioned rats, while it was elevated after further treatment with oe-NMNAT2; besides, treatment of sh-NMNAT2 could decrease the expression of NMNAT2 in the brain tissues of 6-OHDA-lesioned rats receiving FMT (Fig. 6A). The behaviors of rats were observed on the 2nd and 3rd week after 6-OHDA injection. The number of rotations was increased, and the moving distance and speed as well as rod latency were decreased in 6-OHDA-lesioned rats; whereas, these effects were counteracted after further overexpression of NMNAT2. Moreover, NMNAT2 knockdown could increase the number of rotations while reducing moving distance and speed as well as rod latency in 6-OHDA-lesioned rats receiving FMT (Fig. 6B–E).

Moreover, IHC displayed that TH-positive neurons were decreased and NMNAT2 protein expression was diminished in SN of 6-OHDA-lesioned rats, while further treatment with oe-NMNAT2 increased TH-positive neurons and NMNAT2 protein expression (Fig. 6F–G). NMNAT2 knockdown decreased TH-positive neurons and NMNAT2 protein expression in SN of 6-OHDA-lesioned rats receiving FMT. Additionally, it was also revealed that the content of MDA was elevated, and GSH content, GSH-Px activity, and SOD activity were reduced in brain tissues of 6-OHDA-lesioned rats. Meanwhile, these effects were counteracted by further NMNAT2 overexpression. NMNAT2 knockdown elevated the content of MDA and diminished GSH content, GSH-Px activity, and SOD activity in brain tissues of 6-OHDA-lesioned rats receiving FMT (Fig. 6H–K). Thus, upregulation of NMNAT2 suppressed the neurobehavioral deficits and oxidative stress in 6-OHDA-lesioned rats. Furthermore, silencing of NMNAT2 counteracted the effect of FMT on the neurobehavioral deficits and oxidative stress response in 6-OHDA-lesioned rats.

## Discussion

In recent years, emerging evidence has indicated that gut microbial dysbiosis is essential for pathogenesis of PD [36, 37]. The data obtained in our study demonstrated that dysbiosis of gut microbiota promoted neurobehavioral deficits and oxidative stress in 6-OHDA-lesioned rats by reducing NMNAT2 expression.

In the composition and abundance of gut microbiota dysbiosis, both the enteric nervous system and central nervous system can be affected, leading to central nervous system diseases [38, 39]. Based on our findings, the diversity, abundance, and functional composition of gut microbiota clearly differed between PD patients and healthy individuals, which concur with the findings of previous studies on the roles of gut microbiota in PD. They have consistently reported the differences in diversity, composition, and abundance of gut microbiota between normal controls and PD patients [40–42].

Moreover, the current study also demonstrated that dysbiosis of gut microbiota might participate in the occurrence and development of PD through the superpathway of arginine and polyamine biosynthesis and NAD^+^ anabolic pathways. It is also interesting to note that arginine metabolites, including polyamine metabolism, are correlated with neurodegenerative diseases, including PD [43]. Numerous studies have explained the roles of NAD^+^ and related metabolites in the occurrence and progression of PD [34, 44], but the mechanistic basis remains poorly understood. In this study, we demonstrated that NMNAT2, as a key gene related to the NAD^+^ pathway, was identified to be downregulated in the brain tissues of PD patients. Further, overexpression of NMNAT2 alleviated neurobehavioral deficits and oxidative stress in 6-OHDA-lesioned rats. Consistent with our results, a prior study has also reported that NMNAT2 mRNA level is decreased in PD, and upregulated NMNAT2 inhibits the progression of PD [26]. Indeed, the activity of NMNAT2 plays a vital role in axonal survival. Silencing of NMNAT2 induces axonal degeneration, a prominent feature of PD [27, 45], suggesting that overexpression of NMNAT2 may curtail the progression of PD.

Furthermore, the obtained data also revealed that FMT alleviated neurobehavioral deficits and oxidative stress in 6-OHDA-lesioned rats. Gut microbiota is manipulated to influence health, which has recently attracted greater attention in alleviating gut microbiota dysbiosis-associated diseases through FMT [46]. FMT is the introduction of gut microbiota obtained from healthy donors into patients’ gut to restore healthy microbiota and treat diseases [47]. It has been documented to be a promising therapy for *Clostridium difficile* infection and some neurological diseases [48]. Importantly, evidence has demonstrated FMT as a strategy to treat various dysbiosis-related gut diseases by changing gut microbiota more robustly relative to food or probiotics, acting as a novel potential therapeutic strategy for PD [49, 50]. This indicates that FMT may be a potential strategy for alleviating neurobehavioral deficits and oxidative stress response in PD.

## Conclusion

**Fig. 7:**
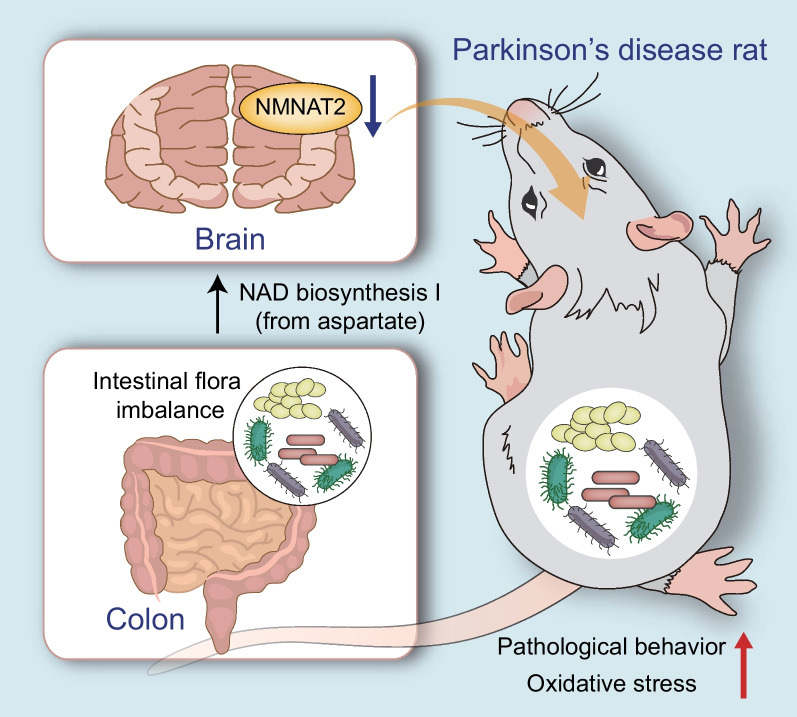
Schematic illustration of the mechanisms of dysbiosis of gut microbiota in PD. Dysbiosis of gut microbiota aggravates neurobehavioral symptoms and oxidative stress responses in 6-OHDA-lesioned PD rat models via regulation of NMNAT2, which is a key gene of the NAD^+^ anabolic pathway

The findings reported here provide evidence supporting that dysbiosis of gut microbiota exacerbated neurobehavioral deficits and oxidative stress in 6-OHDA-lesioned rats by inhibiting NMNAT2 expression, thereby promoting the progression of PD. Furthermore, FMT or restoration of NMNAT2 alleviated neurobehavioral deficits and oxidative stress in 6-OHDA-lesioned rats (Fig. 7). Results of the current study may provide potential clues on identifying strategies to alleviate PD symptoms. Nevertheless, there are still limitations in this study. Firstly, certain difference exists between human gut microbiota and rat gut microbiota, but this study failed to validate the results in human samples. Secondly, this study did not perform the selective transfer of the bacterial species, and it is unclear which bacteria species play a key role. Finally, whether the positive regulation of intestinal microbiota on NMNAT2 is direct or mediated by some intermediate factors remains undefined.

## Data Availability

The datasets generated and/or analyzed during the current study are available in the manuscript and additional materials.

## Abbreviations

PD: Parkinson’s disease
6-OHDA: 6-Hydroxydopamine
SRA: Sequence Read Archive
FMT: Fecal microbiota transplantation
NMNAT2: Nicotinamide mononucleotide adenylyl-transferase 2
NAD +: Nicotinamide adenine dinucleotide
PCoAL: Principal coordinate analysis
LDA: Linear divergence analysis
KEGG: Kyoto Encyclopedia of Genes and Genomes
GEO: Gene Expression Omnibus
SPF: Specific pathogen free
PBS: Phosphate buffered saline
IHC: Immunohistochemistry
ELISA: Enzyme-linked immunosorbent assay
MDA: Malondialdehyde
GSH: Glutathione
SOD: Superoxide dismutase
GSH-Px: Glutathione peroxidase
